# Fears of disclosure and misconceptions regarding domestic violence reporting amongst patients in two US emergency departments

**DOI:** 10.1371/journal.pone.0260467

**Published:** 2021-12-02

**Authors:** Leigh Kimberg, Juan A. Vasquez, Jennifer Sun, Erik Anderson, Clarissa Ferguson, Mireya Arreguin, Robert M. Rodriguez

**Affiliations:** 1 Department of Medicine, University of California, San Francisco, California, United States of America; 2 Department of Emergency Medicine, NYU Langone Health, New York, United States of America; 3 Department of Emergency Medicine, Highland Hospital-Alameda Health System, Oakland, California, United States of America; 4 Department of Emergency Medicine, University of California, San Francisco, California, United States of America; Population Council, INDIA

## Abstract

Patients often do not disclose domestic violence (DV) to healthcare providers in emergency departments and other healthcare settings. Barriers to disclosure may include fears and misconceptions about whether, and under what circumstances, healthcare providers report DV to law enforcement and immigration authorities. We sought to assess undocumented Latino immigrants (UDLI), Latino legal residents/citizens (LLRC) and non-Latino legal residents/citizens (NLRC) beliefs about disclosure of DV victimization to healthcare providers and healthcare provider reporting of DV to law enforcement and immigration authorities. From 10/2018-2/2020, we conducted this survey study at two urban emergency departments (EDs) in California. Participants, enrolled by convenience sampling, responded to survey questions adapted from a previously published survey instrument that was developed to assess undocumented immigrant fears of accessing ED care. Our primary outcomes were the proportions of UDLI, LLRC and NLRC who knew of someone who had experienced DV in the past year, whether these DV victims were afraid to access ED care, reasons DV victims were afraid to access ED care, and rates of misconceptions (defined according to current California law) about the consequences of disclosing DV to healthcare providers. Of 667 patients approached, 531 (80%) agreed to participate: 32% UDLI, 33% LLRC, and 35% NLRC. Of the 27.5% of respondents who knew someone who experienced DV in the past year, 46% stated that the DV victim was afraid to seek ED care; there was no significant difference in this rate between groups. The most common fears reported as barriers to disclosure were fear the doctor would report DV to police (31%) and fear that the person perpetrating DV would find out about the disclosure (30.3%). Contrary to our hypothesis, UDLI had lower rates of misconceptions about healthcare provider and law enforcement responses to DV disclosure than LLRC and NLRC. Fear of disclosing DV and misconceptions about the consequences of disclosure of DV to healthcare providers were common, indicating a need for provider, patient, and community education and changes that lower barriers to help-seeking.

## Introduction

Domestic violence (DV), also called “intimate partner violence”, is a hidden epidemic that results in a myriad of adverse health effects including acute injuries, chronic pain, mental health disorders, increased risk of sexually transmitted infections, and poor overall health [1–3]. In the United States, 1 in 4 women and 1 in 11 men experience sexual or physical violence and/or stalking over their lifetime [3]. Among women patients cared for in emergency departments (EDs), 14%-30% have experienced DV in the past year and 15%-54% have experienced DV in their lifetime [4, 5]. Despite this high prevalence, only one third or fewer women are screened for DV in Eds [6, 7] and only 5.8% or fewer of people experiencing DV victimization are identified [7–9]. People experiencing DV victimization have many reasons not to disclose DV to healthcare providers including fear of the person perpetrating DV, worry about unhelpful actions or reactions from the healthcare provider, shame, and lack of familiarity with laws or helpful resources [10–15]. These barriers are present to a greater degree among immigrant minority women, especially undocumented women or women from mixed documentation status families [16–18]. If people experiencing DV do disclose victimization to healthcare providers, interventions such as brief counseling, personalized safety planning, and referral to advocacy services have been found to improve health and safety [19, 20]. We have previously demonstrated that because of concerns of discovery, deportation and denial of services, undocumented Latino immigrants (UDLI) have heightened fear when accessing emergency care [21]. Misconceptions about physician reporting of DV to police and immigration authorities may similarly deter people experiencing DV from seeking medical care.

In this study of ED patients, we sought to assess undocumented Latino immigrants (UDLI), Latino legal residents/citizens (LLRC) and non-Latino legal residents/citizens (NLRC) beliefs about disclosure of DV victimization to healthcare providers and healthcare provider reporting of DV to law enforcement and immigration authorities. Specifically, we sought to assess whether ED patients knew of people experiencing DV victimization, whether these people were afraid to disclose DV and seek care, and whether UDLI status was associated with greater fear and more misconceptions about the consequences of DV disclosure to healthcare providers.

Our study is the first to examine the association between immigrant documentation status, perceptions about the reasons DV victims fear and avoid accessing medical care, and knowledge about information-sharing amongst healthcare, law enforcement and immigration authorities. Although one study examined the relationship between help-seeking and legal immigration status and found that undocumented Latina woman experiencing DV sought medical care far less frequently than documented Latina women, the specific reasons for not seeking care were not elucidated [22]. Our study is also the first to query patients who identify as undocumented about their knowledge and opinions of a DV mandatory reporting law requiring healthcare providers to report DV injuries to law enforcement. We also examine the accuracy of undocumented and documented patients’ knowledge about a DV mandatory reporting law, decades after it was introduced [23].

## Methods

### Study design and setting

From October 2018 to February 2020, we conducted this survey study at two urban public county hospitals and Level 1 trauma centers in California (Zuckerberg San Francisco General Hospital [San Francisco, CA] and Highland Hospital—Alameda Health System [city of Oakland in Alameda County CA]) with annual ED censuses of 77,000 and 73,000 patients, respectively. At these EDs, 38% of visits were by patients of self-declared Latino ethnicity in 2017. San Francisco is a city and county inhabited by ~880,000 people including 15% Latino residents [24] and ~46,000 undocumented immigrants [25] and Oakland is a city within Alameda county, a county inhabited by ~1,671,000 residents including 22% Latino residents [26] and ~109,000 undocumented immigrants [27].

We obtained institutional review board (IRB) approval from the University of California of San Francisco Committee on Human Research and the Highland Hospital—Alameda Health System IRB to conduct this survey study by scripted verbal consent.

### Survey development

With experts in domestic violence, we adapted a previously published survey instrument that was developed to assess undocumented immigrant fears of accessing ED care [21]. The final version of revised the instrument consisted of 42 yes/no, multiple choice, free text response, and numerical analog questions. It was pilot tested on six UDLI and LLRC. Participants demonstrated excellent understanding and response consistency. The survey, administered verbally, took less than eight minutes on average to complete.

### Participants and enrollment

We enrolled participants by convenience sampling when study personnel were available (usually in four- to six-hour blocks on weekdays). Emergency department tracking boards were screened for eligible adult patients. Exclusion criteria included patients that were: 1) being evaluated for trauma; 2) transferred from another facility; 3) unable to participate in an interview because of intoxication, altered mental status or critical illness; 4) presenting while incarcerated; 5) on a psychiatric hold. Recruitment, enrollment, and survey administration were done privately, without accompanying persons nor healthcare staff present.

After surveys were completed, we categorized participants into three groups (UDLI, LLRC, and NLRC) by their responses to: *Do you identify as being of Latino origin*? and *Are you a legal resident/citizen of the United States (US)*? Our primary outcomes were 1) the proportions of UDLI, LLRC and NLRC who knew of someone who had experienced DV victimization in the past year 2) whether these people who experienced DV victimization were afraid to come to the ED for care; and 3) their perceptions of the reasons that the people they knew who were experiencing DV victimization may have been afraid to come to the ED for care.

In order to enroll approximately similar numbers in the three groups, we examined our central database tally of the participant group assignments quarterly. When the total number of respondents in one group fell to 25% lower than the other groups, we shifted toward approaching more patients in that group. We excluded respondents after interviews if they were vacationing in the US or if they were non-Latino undocumented immigrants.

### Sample size considerations

For a 7% CI around point estimates of our primary outcome questions, we calculated that we would need to enroll 196 participants in each of the three groups for a total of 588 participants. Because of research constraints imposed by the COVID-19 pandemic, we enrolled 528 total participants (167 UDLI, 175 LLRC, 186 NLRC) before stopping enrollment on February 27, 2020. This reduction widened the CIs around point estimates to approximately 7.4%.

### Survey administration

Surveys were administered by physicians, students, and other research personnel, most of whom were fluent in Spanish and received orientation, training sessions and direct observation to ensure standard survey technique and appropriate response in case of DV disclosure. After scripted verbal consent, these personnel read the survey questions to participants in their preferred language in private ED areas. ED registration records were reviewed to determine whether respondents had a social security number (SS#); none of these were recorded to protect patient identity. Once the survey was completed, interviewers assured participants that EDs maintain confidentiality regarding immigration status. Information about local resources for primary medical care, social services, and domestic violence services were offered to all participants.

### Primary outcomes

Our primary outcomes were perceptions about fears that people experiencing DV have about disclosing DV victimization to healthcare providers, misconceptions about physician reporting of DV to police or to immigration authorities, and misconceptions about police reporting of people experiencing DV victimization to immigration authorities. Toward these outcomes, we compared 95% CIs around differences in proportions for key question responses for the UDLI, LLRC and NLRC groups. We also stratified responses according to other patient characteristics, e.g., age, gender, primary language.

Participant “misconceptions” referred to inaccurate perceptions about healthcare provider mandatory reporting of DV to police and physician and police reporting of people experiencing DV victimization and/or perpetration to immigration authorities. This study was conducted in California where healthcare providers are mandated by state law to report DV to law enforcement when patients present with a physical injury that is “known or suspected to be caused by assaultive or abusive conduct”, regardless of patient consent [23]. Reporting uninjured patients to law enforcement is a violation of patient confidentiality, although healthcare providers may offer to assist people experiencing DV victimization in contacting law enforcement and, if the patient desires, requesting that law enforcement come to the ED to respond. Additionally, California and the two cities where this study was conducted (San Francisco and Oakland) have “Sanctuary” laws that place limits upon the collaboration between law enforcement and immigration authorities [28].

### Data management and analysis

Using standard quality control measures including double checks on data entry, we entered data into REDCap hosted by the University of California, San Francisco. We used STATA v 15.1 (StataCorp, College Station, TX) for analyses, summarizing patient characteristics as raw counts and frequency percent and key survey questions as percentages (proportions) with 95% confidence intervals (CIs). Non-responses to questions were not included in the denominators of proportions for those questions.

## Results

### Characteristics of study participants

Of the 667 patients approached, 531 (80%) were consented and agreed to participate. We excluded 2 patients because they were non-Latino undocumented immigrants and one patient because they were only visiting the US briefly. Of the remaining 528 patients 167 (32%) were UDLI, 175 (33%) were LLRC, 186 (35%) were NLRC. On review of ED registration records, 22% of UDLI, 96% LLRC, and 97% NLRC had social security numbers. UDLI and LLRC had similar gender and age characteristics, while NLRC were older and more often male (Table 1).

**Table 1:** Patient characteristics sorted according to study groups.

	UDLI	LLRC	NLRC	All Respondents
	n (%)	n (%)	n (%)	n (%)
**Total Number**	167 (32)	175 (33)	186 (35)	528 (100)
**Female**	77 (46)	80 (46)	75 (40)	232 (44)
**Male**	89 (53)	95 (54)	108 (58)	292 (55)
**Median age in years (IQR)**	40 (31, 50)	39 (27, 55)	51 (36, 60)	43 (31, 56)
**Primary Language** *				
English	3 (2)	61 (35)	161 (87)	225 (43)
Spanish	157 (94)	111 (63)	1 (.05)	269 (51)
Other	7 (4)	3 (2)	23 (12)	33 (6)
**English Proficiency**				
Not at all	30 (18)	6 (3)	0	36 (7)
A little	85 (51)	43 (25)	1 (0.5)	129 (24)
Most of it	37 (22)	28 (16)	15 (8.5)	80 (15)
Completely	15 (9)	98 (56)	170 (91)	283 (54)
**Have Health Insurance**				
Private	3 (2)	5 (3)	12 (7)	20 (4)
Medicare/Medi-Cal	42 (25)	116 (66)	149 (80)	307 (58)
**Healthy SF/HealthPAC** **	49 (29)	12 (7)	8 (4)	69 (13)
**Have Housing**	160 (96)	159 (91)	155 (83)	474 (81)
**Have a PCP**	100 (60)	114 (65)	119 (64)	333 (63)
**If no PCP, where do you get care?**				
Clinic	10 (6)	16 (9)	14 (8)	40 (8)
Emergency Department	40 (60)	29 (48)	41 (22)	110 (21)
Other/none	17 (25)	16 (26)	12 (18)	45 (23)

PCP—primary care provider; IQR—interquartile range; UDLI—Undocumented Latino Immigrant; LLRC—Latino Legal Resident/Citizen; NLRC—Non-Latino Resident/Citizen.

*Patients could declare more than one primary language

**Healthy SF and HealthPAC are Department of Public Health programs in San Francisco and Alameda counties that provide free or affordable health services to those who are not eligible for public insurance (Medi-Cal and Medicare) and are below certain income threshold.

Most (67%) of the UDLI participants had been living in the US for more than 10 years and 14% stated that they were brought to the US as children; the vast majority (94%) had Spanish as their primary language and 69% reported little to no English proficiency; UDLI were less likely to have health insurance (UDLI 62.9% vs LLRC 82.3%: difference 19.4%, 95% CI 10.0–28.4%; UDLI 62.9% vs NLRC 86.0%: difference 23.2%, 95% CI 14.1–31.9%). Eighty-two percent of UDLI had seen a physician in the US previously; sixty percent of UDLI had a primary care physician but the majority (60%) of UDLI accessed care through the ED.

Few UDLI (13.8%), LLRC (18.9%) and NLRC (17.2%) believed that ED providers treat patients differently based on legal status, and few UDLI (5.4%), LLRC (12%) and NLRC (13.9%) believed that healthcare providers report non-US citizens/residents to immigration. More UDLI reported feeling “a lot” or “some” worry that themselves, a family member, or a close friend could be deported as compared to NLRC (UDLI 25.2% vs NLRC 11.8%: difference 13.3, 95% CI 5.3–21.4%) (Table 2).

**Table 2:** Responses to key questions sorted according to study groups.

	UDLI	LLRC	NLRC	All
	% (95% CI)	% (95% CI)	% (95% CI)	% (95% CI)
**Q1. Do you believe that doctors and nurses treat US citizens/residents differently than non-US citizens/residents?**				
Yes	14 (9–19)	19 (13–25)	17 (12–23)	17 (13–20)
No	77 (7–82)	65 (61–75)	58 (50–64)	68 (63–71)
Unsure	9 (5–14)	12 (7–17)	25 (19–31)	16 (12–19)
**Q2. Before this interview, did you believe that doctors and nurses report non-US citizens/residents to the immigration authorities?**				
Yes	6 (2–9)	12 (7–17)	14 (9–19)	11 (8–13)
No	75 (67–80)	69 (61–74)	49 (41–56)	64 (59–67)
Unsure	20 (14–26)	19 (14–25)	35 (28–42)	25 (21–28)
**Q3. Regardless of your immigration or citizenship status, how much, if at all, do you worry that you, a family member, or a close friend could be deported?**				
A lot	22 (16–29)	15 (10–20)	4 (2–8)	13 (10–16)
Some	3 (1–6)	8 (4–12)	8 (4–12)	6 (4–8)
Not much	3 (1–6)	6 (3–10)	7 (3–10)	5 (3–7)
Not at all	7 (4–12)	13 (8–18)	27 (21–33)	16 (13–19)
**Q4. Do you know someone (yourself, family, friend, other) who has experienced domestic violence in the past year?**				
Yes	19 (13–25)	33 (26–39)	30 (24–37)	28 (23–31)
No	78 (71–84)	66 (59–72)	63 (56–70)	69 (65–72)
**Q5. Were any of the people you know who experienced domestic violence in the past year afraid to go to the doctor?**				
Yes	39 (23–56)	54 (41–66)	42 (30–55)	46 (38–54)
No	48 (31–65)	39 (27–51)	51 (38–63)	46 (38–54)
Unsure	10 (3–24)	5 (1–14)	7 (1–14)	7 (3–12)
**Q6. Did the person(s) you know who experienced domestic violence and was afraid go to the doctor anyway?**				
Yes	66 (39–86)	45 (29–62)	59 (41–75)	54 (42–65)
No	25 (9–53)	45 (29–62)	38 (23–56)	39 (28–50)
Unsure		10 (3–25)		4 (1–12)
**Q7. Do you believe that doctors report domestic violence to the police?**				
Yes, always	16 (10–21)	33 (26–40)	36 (28–42)	28 (24–32)
Yes, but only when there is physical injury	40 (32–47)	34 (27–41)	32 (25–39)	35 (31–39)
No, never	17 (12–23)	10 (6–15)	7 (4–11)	11 (8–14)
Unsure	26 (20–33)	22 (16–28)	22 (16–28)	23 (19–27)
**Q8. Do you believe doctors report** **victims** **of domestic violence who are undocumented to immigration authorities?**				
Yes	9 (5–14)	17 (12–23)	28 (22–34)	18 (15–21)
No	56 (49–64)	50 (42–57)	33 (26–40)	47 (42–50)
Unsure	34 (26–40)	31 (25–38)	36 (28–42)	34 (29–37)
**Q9. Do you believe that the police report** **victims** **of domestic violence who are undocumented (non-US citizens) to immigration authorities?**				
Yes	32 (25–39)	41 (34–48)	51 (43–57)	42 (37–45)
No	38 (30–45)	33 (26–39)	20 (15–26)	30 (26–33)
Unsure	29 (22–36)	25 (19–32)	25 (19–31)	26 (22–30)
**Q10. Do you believe that doctors** **should** **report domestic violence to the police even when the victim does not want the police to find out about the domestic violence?**				
Yes, I strong think they should	50 (42–57)	47 (40–54)	40 (33–46)	46 (41–49)
Yes, I somewhat think they should	22 (16–28)	18 (13–24)	23 (17–29)	21 (17–24)
Unsure	13 (8–18)	11 (7–16)	10 (6–14)	11 (8–13)
No, I strongly think they should not	12 (7–17)	17 (11–22)	17 (12–22)	15 (12–18)
No, I somewhat think they should not	4 (2–8)	6 (3–10)	7 (4–11)	6 (4–8)

UDLI—Undocumented Latino immigrant; LLRC—Latino legal resident/citizen; NLRC—Non-Latino resident/citizen; CI—confidence interval.

### Main outcomes

Approximately one-quarter (27.5%, 95% CI 23.8–31.4) of all the respondents stated they knew someone who experienced DV in the past year. Of those who knew someone who had experienced DV, 46% reported the person experiencing DV victimization was afraid to seek medical care after the DV; differences in proportions did not differ significantly between groups. UDLI patients reported that the person they knew who was experiencing DV would be fearful of going to see a doctor because they felt ashamed (29%), feared that the doctor would report DV to the immigration authorities (29%), and feared that the perpetrator would find out about the disclosure (26%). The primary fears reported by LLRC were fear the perpetrator would find out about the disclosure (39%), fear the doctor would report DV to police (35%), and fear the perpetrator would be deported (28%). The primary reasons NLRC reported were responsible for fear of DV victimization disclosure were fear the doctor would report DV to the police (32%), fear the perpetrator would find out about the disclosure (25%), and feeling ashamed (23%). Although the rank order of fears varied, there were no significant differences between groups in these proportions. Seventeen percent of participants reported that the people they knew who had experienced DV feared that their children would be taken away from the family. Of the participants who knew someone who experienced DV in the past year *and* reported the person experiencing DV victimization was afraid to seek medical care, 39% reported that the person experiencing DV victimization did not seek care due to fear.

Misconceptions and uncertainty about when physicians in CA report DV victimization to the police were common. Only 40.1% ULDI, 33.7% LLRC, and 32.3% NLRC correctly understood that CA law mandates that healthcare providers report to police only when the patient experiencing DV has physical injury. Fewer ULDI had misconceptions than NLRC about physician (UDLI 8.9% vs NLRC 27.9%; difference 19.4%, 95% CI 11.6–26.9) and police (UDLI 31.8% vs NLRC 50.5%; difference 18.8%, 95% CI 10.1–29.8) reporting of undocumented people experiencing DV to immigration authorities. Misconceptions about physician or police reporting of undocumented people experiencing DV victimization to immigration authorities did not differ significantly between the UDLI and LLRC groups (Table 2).

Most participants UDLI (71%), LLRC (66%), NLRC (63%) responded “Yes, strongly” or “Yes, somewhat” when asked whether doctors *should* report DV to police even if the person experiencing DV victimization does not want the police notified. When stratified by male and female gender, 66% of men and 68% of women responded “Yes, strongly” or “Yes, somewhat” to the same question.

## Discussion

The provision of counseling, personalized safety planning, and follow-up advocacy referrals to patients who disclose DV to healthcare providers improves mental health consequences, the use of safety behaviors and reduces subsequent violence [19, 20]. Yet, DV screening rates by healthcare providers and DV disclosure rates by patients are low [6–9]. In this study of UDLI, LLRC and NLRC perceptions about the barriers and consequences of people experiencing DV victimization disclosing DV to physicians, we found that ED patients believed that the people they knew who were experiencing DV were fearful about seeking medical care and avoided medical care due to fear. Contrary to our initial hypothesis, the UDLI group had fewer misconceptions about the consequences of DV disclosure to healthcare providers.

Fear that the doctor would report DV to the police was the most common barrier to seeking care identified by our study participants. In a study conducted by the National DV Hotline, fear about calling the police was extremely common; 80% of people experiencing DV victimization who had never previously called the police and 66% of people who had called the police previously were fearful of calling. After interacting with the police, 1/5 of people experiencing DV victimization felt safer, 1/3 felt less safe, 1/2 felt like it made no difference in their safety, and 2/5 felt that the police had discriminated against them based on gender, race and other factors [29, 30]. Law enforcement response to DV does not always result in an arrest or confiscation of firearms and may have dangerous consequences for people being victimized, including the arrest of the person being victimized instead of or in addition to the person perpetrating the DV (“dual arrest”); lack of English proficiency increases the risk that a person being victimized is arrested [31–33]. Law enforcement involvement is likely to alert the person perpetrating DV that the person they are harming may have disclosed the DV to others; this can result in an escalation of violence. Additionally, Latino community members perceive law enforcement officers’ lack of sensitivity and insufficient knowledge about the complexity of DV situations and how best to protect people experiencing victimization as significant barriers to improving safety [16].

These findings have raised concerns that laws mandating reporting of DV to law enforcement may, in fact, discourage people experiencing DV victimization from seeking help [15, 34–36]. Women enrolled in a health maintenance organization had complex and seemingly contradictory opinions about mandatory reporting of DV to police, believing that it would make it easier to find help yet would dissuade women from disclosing abuse to healthcare providers and put them at greater risk of future abuse [10]. While many states have laws mandating that healthcare providers report injuries resulting from weapons, crimes, violence or intentional acts to law enforcement, the benefits of such laws are unclear [15, 34, 35, 37, 38]. Misconceptions about when physicians must report DV victimization to police were common amongst all of the ED patients (ULDI, LLRC and NLRC) in our study. As in other studies, despite the risk of harm, lack of proven efficacy, and high rates of dissatisfaction associated with police involvement in DV, 67% of our study participants thought that DV should be reported to law enforcement without patient consent [11, 16, 35, 39].

Patients who knew someone experiencing DV victimization reported that fear that the person perpetrating the DV would find out about the DV disclosure (31%) and/or feeling ashamed (23%) were barriers to seeking care; these fears are common amongst diverse communities. Women from the San Francisco Bay Area who had experienced DV victimization identified the stigma associated with experiencing DV victimization, feeling responsible for their abuse, and feeling guilty for staying in an abusive relationship as barriers to seeking help from healthcare providers [31]. Cultural norms in the Latinx (and other) communities may result in people experiencing DV victimization protecting the family image at the expense of their own needs and avoiding bringing shame to the family by keeping the abuse a secret [16]. Because family and friends may alert the person perpetrating the violence about DV disclosure, healthcare providers must not discuss DV with family or friends present and should always use professional interpreters to discuss DV with patients who have limited English proficiency [40, 41]. Women experiencing DV victimization report that healthcare providers are most helpful when they are compassionate, nonjudgmental, inquire about DV but do not push for disclosure, protect confidentiality, demonstrate understanding that DV is a complex and challenging problem that is unlikely to be resolved quickly and requires a patient-centered, personalized, and non-directive approach [42], and offer tangible supportive resources [42, 43]. Expressing respect for the autonomy [43, 44] and remarkable survival skills of people experiencing DV victimization can reduce stigma and shame.

Participants perceived the fear of losing custody of children (17%) as a reason for not seeking medical care after DV. The perpetration of DV forces parents, (most often mothers), who are being victimized to make exceedingly complex decisions to survive and protect their children, especially when they lack access to money, employment, food, and housing. Mothers experiencing DV victimization often strategize about disclosure by prioritizing their own safety and the physical safety and emotional well-being of their children [35]. Their decision-making about whether to stay in or leave an abusive relationship is often linked to fear of losing custody of their children and to feeling responsible for their children’s safety [12, 13, 45]. Mexican immigrant mothers living in New York City who became involved with public child welfare services because of DV, often felt like a “bad mother,” who had failed to protect their children, and “bad women” who turned family members over for deportation [45]. When asked about turning to state agencies or the police for help, they expressed desire for the state to protect their rights as women, but not to violate their rights as mothers [46].

In prior studies, immigrant Latina women have expressed the belief that healthcare providers would report them to immigration authorities and that fear of deportation prevents undocumented people experiencing DV victimization from disclosing DV to healthcare providers [16, 47, 48]. In our study, which was done in two cities with well-publicized Sanctuary city laws, only 11% of ED patients thought that physicians and nurses report undocumented patients to immigration authorities; no differences in this perception were found amongst UDLI, LLRC and NLRC. ED patients were much less certain about whether physicians report undocumented people experiencing DV victimization to immigration authorities; one third of ED patients were unsure about this. Only 9% of UDLI thought that physicians report people experiencing DV victimization to immigration authorities, significantly less than the 28% of NLRC who thought that physicians do report people experiencing DV to immigration authorities. There were no significant differences between UDLI and LLRC.

Our study participants reported that the people they knew who were experiencing DV victimization were reluctant to seek care due to fear that the police would report them (17%) or the person perpetrating the DV (19%) to immigration authorities. This is a commonly reported fear and barrier to help-seeking. Community awareness of immigration enforcement was found in one US city to reduce calls to law enforcement for assistance with DV [49]. In Miami, a focus group study that included people who had experienced DV, community members, and service providers, found that immigrants were perceived as being most vulnerable to DV; participants highlighted that fear of being deported or having their partner deported prevents help-seeking [16]. In prior studies, undocumented Latina women with limited English proficiency have been found to be unfamiliar with laws preventing deportation of people experiencing DV victimization [16, 50, 51]. In contrast, we found that fewer UDLI had misconceptions about physician and police reporting to immigration authorities than NLRC. We found that misconceptions about police reporting of people experiencing DV victimization to immigration authorities were quite a bit more prevalent than misconceptions about physician reporting of DV to immigration authorities; anti-immigration statements made by politicians [21], publicity about law enforcement cooperation with immigration authorities in non-Sanctuary locations [49], and instances of law enforcement cooperation with immigration authorities in Sanctuary locations likely seed these beliefs [52].

DV, especially when compounded by structural violence, is an exceedingly complex and harmful phenomenon; neither the healthcare system nor society have developed sufficiently efficacious and personalized interventions to decrease violence and improve safety and well-being. Our study participants reported that the fears of people experiencing DV prevented access to healthcare; they also held misconceptions about the consequences of DV disclosure. What can be done to reduce fear, rectify misconceptions, and improve care? In one of our cities (SF), after a history of unsuccessful advocacy to repeal or modify the California DV mandatory reporting law to support patient confidentiality and autonomy [53, 54], we worked with local law enforcement to develop a supplemental form to the California mandatory healthcare report to allow people experiencing DV to provide input to law enforcement about their own assessment of how to minimize safety risks associated with mandatory reporting [55]. Other evidence-based innovations could be deployed; for example, in an ED, the installation of a patient-facing, confidential computer-based health education tool that provides people experiencing DV (victimization and perpetration) with the opportunity to disclose DV and receive educational material, resulted in high levels of DV disclosure and improved rates of documentation of DV in the medical record [56]. DV experts also recommend that all healthcare providers adopt a new approach called “Universal Education” to provide all patients with DV education and resources prior to DV screening to highlight the impacts of DV on health, reduce misconceptions, destigmatize help-seeking, and share confidential options for assistance regardless of disclosure [43].

Partnering with community-based organizations to closely collaborate [20, 57] or, preferably, integrating confidential DV Advocates [58, 59] and Legal Assistance experts [60, 61] from community-based organization into healthcare settings are exceptionally promising practices to improve healthcare provider response, increase referrals, improve safety, reduce violence and the cost of healthcare [58, 59, 62, 63]. Even if ED’s cannot co-locate DV Advocates, immediate referral to social work and offering the patient a private place to call a DV Advocate during the visit can reduce barriers to help-seeking. By acknowledging the fears, misconceptions, dangers, and complexity of this complex hidden epidemic and improving healthcare-based access to DV education and resources, ED’s can contribute to improving the safety and well-being of people experiencing DV and their children. Healthcare providers can also advocate for societal policies, like Sanctuary laws, that reduce the incidence of DV homicides of Latina women [28].

### Limitations

Perhaps the greatest limitation of our work is our inability to discern which participants had personal experience of DV victimization (or perpetration). Moreover, given the secondhand nature of some of our questions, we could not confirm that respondents truly knew people experiencing DV victimization, what number of people experiencing DV victimization they were referring to, the immigration status of the person experiencing DV, and whether their responses regarding the fears of these people were accurate. Nevertheless, other elements of our survey are firsthand reports and likely accurately reflect respondents’ perceptions and misconceptions about DV reporting to police and immigration authorities. Qualitative data would provide further insight into the results, especially given our unexpected finding of lower levels of misconceptions amongst UDLI about both physician and police reporting to immigration authorities. Our results may not be generalizable to patient populations seeking care in other locations, especially in ED’s located in areas that do not have Sanctuary policies. Additionally, our findings may not be generalizable to communities with different racial/ethnic backgrounds or with different levels of English language proficiency. Although we used a convenience sampling method, it is unlikely that 24/7 sampling would significantly change our findings.

## Conclusion

In summary, we found that among patients who knew someone experiencing DV victimization, fear of seeking care was common; the most common fears were the fear of police involvement and that the person perpetrating DV would find out about the DV disclosure. We also found a high prevalence of misconceptions and poor understanding (unsure responses) about the consequences of disclosing DV to healthcare providers and physician and police reporting of DV to law enforcement and immigration authorities. Contrary to our hypothesis, UDLI had lower rates of misconceptions than LLRC and NLRC. Despite acknowledgment of fear of police involvement as a deterrent to seeking medical care, participants expressed high levels of support for mandatory reporting to law enforcement without patient consent, a process that diminishes the autonomy of the person being victimized and has not been proven to prevent nor effectively mitigate adverse outcomes associated with DV. We have identified fears about disclosing DV and knowledge gaps about the consequences of DV disclosure that must be addressed promptly with widespread informational programs to inform communities and dispel myths. Given these fears and misperceptions, the extremely high prevalence of DV amongst patients in ED’s, and the proven benefits of DV advocacy we recommend that ED’s partner more closely with DV advocacy and legal aid organizations and provide all patients with education about DV and information about how to access life-saving community-based and on-site DV advocacy assistance regardless of DV disclosure.

## Supporting information

S1 Dataset (XLSX)
